# No clinical difference at mid-term follow-up between TiN-coated versus uncoated cemented mobile-bearing total knee arthroplasty: a matched cohort study

**DOI:** 10.1051/sicotj/2023001

**Published:** 2023-02-09

**Authors:** Etienne Deroche, Cécile Batailler, Jobe Shatrov, Stanislas Gunst, Elvire Servien, Sébastien Lustig

**Affiliations:** 1 Orthopaedic Surgery and Sports Medicine Department, FIFA Medical Center of Excellence, Croix-Rousse Hospital, Lyon University Hospital 69004 Lyon France; 2 Sydney Orthopaedic Research Institute (SORI) at Landmark Orthopaedics 500 Pacific Hwy St. Leonards NSW Australia; 3 LIBM – EA 7424, Interuniversity Laboratory of Biology of Mobility, Claude Bernard Lyon 1 University 69622 Lyon France; 4 University of Lyon, Claude Bernard Lyon 1 University, IFSTTAR, LBMC UMR_T9406 69622 Lyon France

**Keywords:** Knee arthroplasty, Metal allergy, Coated implants, Mobile bearing, Implant survival

## Abstract

*Introduction*: Nitride-based ceramic coating was introduced into surgical implants to improve hardness, reduce abrasion, and decrease the risk of metal-induced adverse reactions, especially for patients with suspected or identified metal hypersensitivity. The study aimed to evaluate the effectiveness and safety of a titanium nitride (TiN) coated prosthesis with a mobile bearing design. *Methods*: This was a retrospective matched-cohort study from a single center, comparing clinical outcomes between patients receiving either a TiN-coated versus an uncoated cobalt-chromium-molybdenum (CoCrMo) prostheses for primary total knee replacement. Seventeen patients received the TiN prosthesis between 2015 and 2019. These were matched 1:2 with patients receiving uncoated mobile-bearing knee prostheses with the same design manufacturer. *Results*: Fourteen patients in the TiN group had complete 5-year follow-up data and were compared with 34 patients from the CoCrMo group. The Knee Society Score was 170.6 ± 28.0 (Function subscore 83.7 ± 17.5 and Knee subscore 86.9 ± 13.8) in the TiN group and 180.7 ± 49.4 (Function subscore 87.5 ± 14.3 and Knee subscore 93.2 ± 9.6) in CoCrMo group, with no statistically significant difference (*p* = 0.19). One patient underwent a revision for instability requiring the removal of the implant in the TiN group and none in the CoCrMo group. The survival rates were 92.9% (CI95% 77.3–100.0) and 100.0% in the TiN group and CoCrMo group respectively (*p* = 1.0). *Discussion*: TiN-coated TKA with mobile bearing resulted in satisfactory clinical outcomes, and a low revision rate, and there was no complication related to the coated implant. The use of TiN-coated prostheses in case of confirmed or suspected metal allergy provides satisfactory short-term clinic outcomes.

## Introduction

Femoral and tibial components of most total knee prostheses are made of a cobalt–chromium–molybdenum (CoCrMo) alloy. These metal-bearing components have a surface area largely exposed to surrounding soft tissues and are subjected to wear-, corrosion-, and wear-induced corrosion mechanisms that lead to metal nanoparticles and ion release [1]. Increased serum cobalt (Co) and chromium (Cr) levels have been observed following TKA when compared to preoperative levels [2, 3]. Debris from CoCrMo components is cytotoxic in a dose-dependent manner; triggering osteolysis and metal-related sequelae [4–6].

Titanium nitride (TiN)-based ceramic coating was introduced in surgical implants with the intention of enhancing the mechanical properties and biocompatibility of knee components [7–10]. The coating process covers the metal component with a thin ceramic layer, hardening the metal-bearing surface. This protects the prosthesis from abrasion and scratches [11], with the intention of enhancing biocompatibility and reducing metal ion release [12–15].

Despite the proposed advantages of coated implants, evidence of their effectiveness against metal-induced adverse reactions is limited, with inconsistent results. Interpreting the influence of component material on postoperative pain and other clinical symptoms is difficult. Previous studies have reported good to excellent mid-term results for ceramic-coated knee prostheses in primary TKA with survival rates comparable to benchmark survival rates of commonly used implants [9, 15–18]. However, some ex vivo retrieval studies have recently reported signs of third-body wear, delamination, and scratching on coated knee components, raising concerns about the long-term survival of these implants [19, 20]. Due to the limited evidence and lack of comparative studies, the effectiveness and outcomes of ceramic-coated knee implants in primary TKA remain controversial.

Implant design with a low constraint such as a cruciate substituting (CS) (ultra-congruent, deep dished, lipped liner) mobile bearing insert theoretically offers stability through a highly conforming articulation and raised anterior and posterior lips [21].

This study aimed to compare the clinical outcomes of TiN-coated and uncoated CoCrMo mobile-bearing CS knee prostheses in primary TKA, using implant survival as the primary endpoint. Secondary outcomes, including clinical scores and complication rates were also investigated. The hypothesis was that TiN-coated implants can be used in primary mobile-bearing TKA without compromising outcomes compared to standard uncoated CoCrMo implants.

## Methods

**Figure 1:**
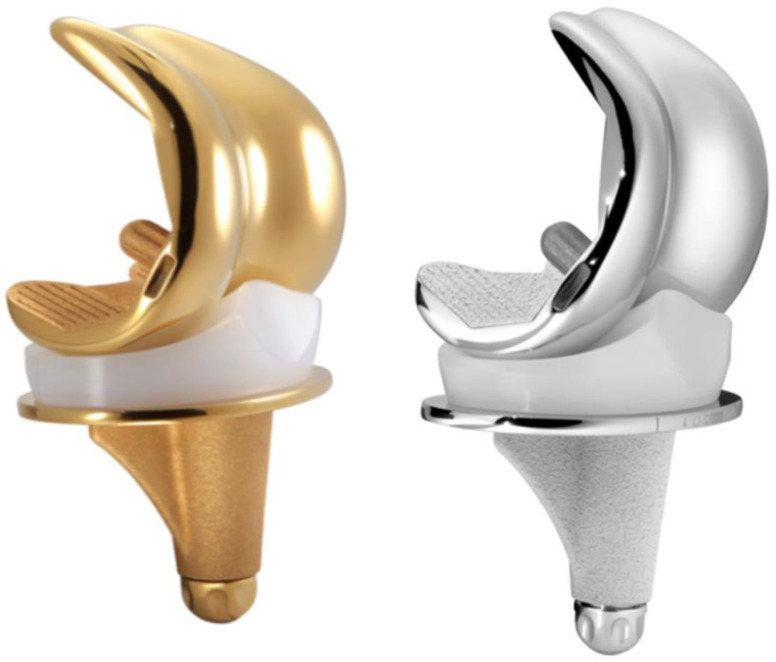
TiN-coated (Score AS) and uncoated CoCrMo (Score II) mobile bearing prostheses (*reproduced with permission from* www.amplitude-ortho.com).

This was a retrospective, single-center comparative study of consecutive patients who underwent primary cemented TKA from the same manufacturer (Amplitude®, Valence 26000, France), between January 2015 and September 2019. Data were prospectively collected in an institutional arthroplasty registry (CliniRecord, Amplitude®). Inclusion criteria were patients undergoing TKA for primary or secondary osteoarthritis. All patients received a CS prosthesis with a mobile-bearing polyethylene insert (Figure 1), with or without patellar resurfacing, during the study period. Exclusion criteria were patients undergoing revision TKA and patients without complete follow-up data at a minimum of 3 years follow-up.

All TKA was performed by a senior surgeon with either TiN-coated (Score Allergy Solution) or an uncoated CoCrMo (Score II) mobile-bearing CS knee prostheses in primary TKA.

Seventeen patients received a TiN-coated prosthesis during the study period and were matched with patients who received a CoCrMo prosthesis with a 1:2 ratio. In all cases, TiN-coated prostheses were chosen based on clinical suspicion of metal allergy. Seventeen patients received a TiN-coated TKA: no patients passed away during the follow-up periods, two were lost to follow-up, and one was excluded due to it being a revision, leaving 14 patients for analysis. All patients reported dermatitis with jewelry, jean buttons, zippers, or after receiving an implantable device. Ten patients returned a positive metal allergy test prior to surgery (Table 1).

**Table 1:** Results of metal allergy tests.

Allergy test (patch skin tests)	Yes: *n* = 10
	No: *n* = 4
Results of allergy test (metal)	Cobalt: *n* = 3
	Nickel: *n* = 5
	Chrome + Cobalt: *n* = 2

**Figure 2:**
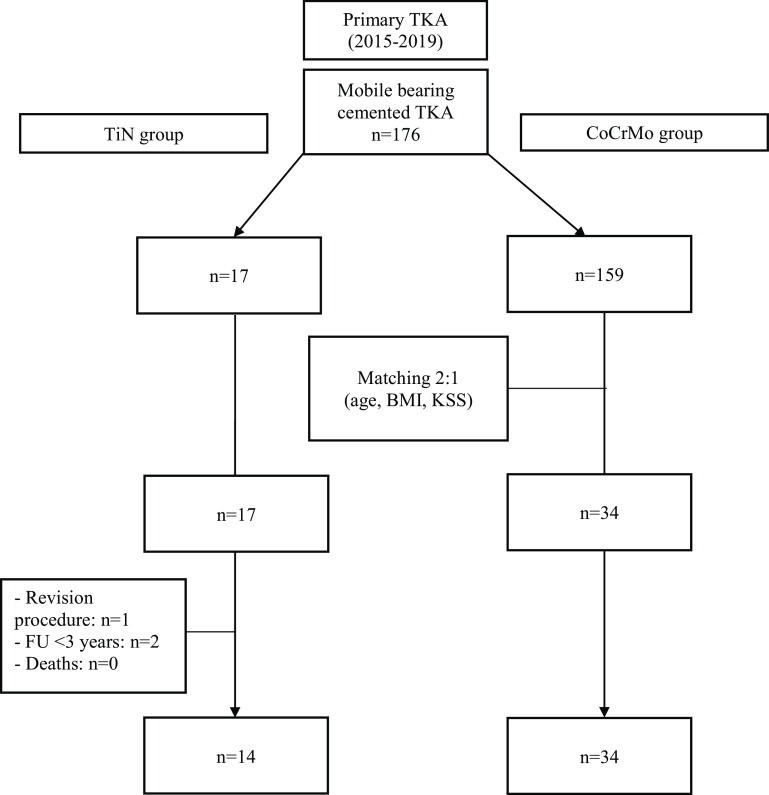
Flowchart of the study.

In the control group, no patients passed away, and all patients completed a 3-year follow-up, leaving 34 patients with a CoCrMo mobile-bearing CS knee prosthesis for analysis. A complete flowchart summarizing patient selection is illustrated in Figure 2. Both groups were similar for all characteristics, except for patellar resurfacing which was routinely performed for the TiN group, as reported in Table 2.

**Table 2:** Characteristics of the groups before surgery.

Characteristics	TiN group (*n* = 14)		CoCrMo group (*n* = 34)		*p*-Value
	Mean ± SD	Min–Max	Mean ± SD	Min–Max	
Age (years)	69.4 ± 6.2		70.9 ± 9.2		0.37
Sex					0.18
Male	*n* = 2 (14%)		*n* = 12 (35%)		
Female	*n* = 12 (86%)		*n* = 22 (65%)		
Body Mass Index (kg/m^2^)	30.4 ± 6.0	21–40	29.0 ± 5.3	19–41	0.44
Side					
Right	*n* = 5 (36%)		*n* = 20 (59%)		
Left	*n* = 9 (64%)		*n* = 14 (41%)		
Hip Knee Ankle angle (°)	178 ± 8	165–191	175 ± 8	162–201	0.2
Approach	Medial: *n* = 10 (71%)		Medial: *n* = 28 (82%)		0.12
	Lateral: *n* = 4 (29%)		Lateral: *n* = 6 (18%)		
*Indications for TKA*					
Medial OA	*n* = 9 (64%)		*n* = 28 (82%)		
Lateral OA	*n* = 2 (14%)		*n* = 6 (18%)		
PF OA	*n* = 2 (14%)		0		
Post-traumatic	*n* = 1 (7%)		*n* = 3 (9%)		
History of HTO	*n* = 1 (7%)		0		
*Surgery*					
Tibial stem	*n* = 2 (14%)		*n* = 6 (18%)		1.0
Patellar resurfacing	*n* = 14 (100%)		*n* = 14 (41%)		**<0.001**
*KSS total*	130.9 ± 35.2	82–184	124.7 ± 38.7	35-230	0.43
*KSFS*	65.4 ± 17.7	45–90	65.4 ± 16.9	0–90	0.38
*KSKS*	65.5 ± 17.3	37–94	59.3 ± 12.2	35-85	0.24
*Maximum flexion*	120 ± 9	95–135	120 ± 11	95-145	0.99

Bold value = statistically significant (*p* < 0.05).

### Surgery

All surgeries were performed without a tourniquet. Patients in the control group received a CoCrMo deep-dish, mobile-bearing TKA (Score II). Patients in the TiN group received a deep-dish, mobile-bearing TKA (Score AS). Both prostheses were produced by the same manufacturer (Amplitude®, Valence 26000, France). With the exception of the surface coating, the two prostheses were identical. A medial sub-vastus approach was used if the pre-operative alignment was in the varus and a lateral parapatellar approach for cases with valgus alignment. Surgery was performed using manual instrumentation and a measured resection technique. Sizing for the femur was done by posterior referencing. All femoral components were referenced from the posterior femoral condyle. External femoral rotation of 3° relative to the posterior condylar axis (PCA) was performed for valgus aligned knees. All other knees had femoral components implanted in neutral rotation relative to the PCA. Balancing of gaps in extension and flexion was assessed manually after osteophyte clearance and removal of the PCL with spacers, and soft tissue releases were performed as required. All components in both groups were cemented and the patella was selectively resurfaced.

### Titanium nitride coating

TiN ceramic surface coating on metallic implants has been employed in the US since the early 1980s, and their use has been steadily increasing in Europe since the early 1990s. The ceramic coating technique utilizes a technology known as physical vapor deposition, which involves coating the implant during the vapor phase in a high-vacuum chamber to which nitrogen is added. During this process, the coating is attached in several layers to the implant surface (Table 3).

**Table 3:** TiN coating characteristics.

Coating thickness	Approximately 4 µm
	Coating thickness is measured using a process known as calotte grinding test, on test pieces that are coated with each implant batch.
Hardness	Ca. 2400 HV (0.1 N) Hardness is measured using a micro-hardness test. The hardness of CoCrMo alloys is only 650 HV (0.1N).
Adhesive strength	Adhesive strength 1–2.
	Adhesive strength is tested in accordance with VDI guideline 3824 using the Rockwell HRC test. In addition, a thorn bending test is carried out using a Scratch test plate. These tests have demonstrated that the coating has outstanding adhesive properties.
Roughness	Ra < 0.05 µm
	Roughness determined with the profile method acc. to DIN EN ISO 4287. These roughness values are in compliance with the DIN EN ISO 21534.
Tribolgoy and wear resistance	Low friction coefficient in contact with UHMWPE; ion release is suppressed upon exposure to frictional fretting. A significantly higher degree of surface scratch resistance.

### Clinical assessment

All patients underwent standardized follow-ups at 2, 12 months, and annually thereafter. Patient-reported outcomes were assessed with the International Knee Society Score (KSS) [22]. Patient satisfaction was assessed using a global clinical outcome measurement and categorized as: very satisfied, satisfied, disappointed, or dissatisfied. The range of motion was recorded using a hand-held goniometer. The complication rate was evaluated at the last follow-up and included all surgical reinterventions (component exchange, debridement, irrigation, mobilization under anesthesia and arthrolysis).

All patients had a pre-operative and postoperative radiographic assessment at 2, 12 months and annually which included: anteroposterior, lateral, weight-bearing, patellar axial, and standing full-length radiographs. Axial views were performed using the Merchant method [23]. Radiolucent lines at the bone-cement interface were considered pathological when >2 mm or evolutive on two consecutive radiographs.

### Statistical analysis

Statistical analyses were performed using MedisticaⒸ, https://www.pvalue.io (*graphic user interface to the R statistical analysis software 2019–22)* with an *α* level set to 0.05. In order to reduce confounding bias, exact matching without replacement was performed to generate similar patient cohorts. Patients with TiN-coated TKA were matched at a 1:2 ratio with controls (CoCrMo-coated TKA) based on the following parameters: age, BMI, and preoperative KSS. Categorical variables were compared with a chi-square test, and continuous variables were compared with Welch’s *t*-test or the Mann–Whitney U test. Survival analysis was performed by the Kaplan–Meier method.

## Results

The mean follow-up was 67 ± 6 months in the TiN group and 39 ± 2 months in the CoCrMo group (*p* < 0.001). Postoperative clinical scores and complications are reported in Table 4. Clinical scores were slightly higher in the CoCrMo group but the difference was not statistically significant. The satisfaction rate and the rate of complications were also comparable, without significant statistical differences.

**Table 4:** Postoperative results and complications.

Postoperative Clinical Data	TiN group (*n* = 14)		CoCrMo group (*n* = 34)		*p*
	Mean ± SD	Min–Max	Mean ± SD	Min-Max	
KSS total	170.6 ± 28.0	39–200	180.7 ± 49.4	61–200	0.19
KSFS	83.7 ± 17.5	10–100	87.5 ± 14.3	30–100	0.45
KSKS	86.9 ± 13.8	38–100	93.2 ± 9.6	31–100	0.09
Maximum flexion	120 ± 12	70–145	122 ± 11	95–140	0.061
Improvement KSS total	39.7 ± 26.0		56.0		0.16
Improvement KSFS	18.3 ± 24.2		22.1 ± 25.8		0.69
Improvement KSKS	21.4 ± 27.1		33.9 ± 38.4		0.25
Very satisfied or satisfied	*n* = 12 (85.7%)		*n* = 31 (91.2%)		0.63
Complications					
Reoperation without implant removal	*n* = 1 (7.1%) Arthroscopic arthrolysis (delay 6 months)		0		1.0
Revision surgery	*n* = 1 (7.1%) TKA revision for instability (delay 22 months)		0		

KSS: Knee Society Score; KSFS: Knee Society Function Subscore; Knee Society Knee Subscore Primary TKA.

The survival rate in the CoCrMo group was 100.0% as there was no revision and no reoperations. In the TiN group, the survival rate was 92.9% (95% CI [77.3–100.0]), *p* = 1.00. In the TiN group, one TKA was revised after 22 months because of instability, requiring a semi-constrained prosthesis (condylar constrained with fixed-bearing polyethylene). Another reoperation was needed for arthroscopic arthrolysis without implant removal after 6 months for stiffness in flexion with a good final result (120° of flexion at the last follow-up). In the CoCrMo group, three patients (8.8%) had nonprogressive radiolucent lines, and one in the TiN group (7.1%, *p* = 1.0). There was no sign of loosening in either group.

## Discussion

The main finding of this study was comparable survival rates between ceramic-coated and uncoated implants after primary TKA at short- to mid-term follow-up. Clinical scores (Knee Society Function subscore and Knee subscore) and complication rates were also comparable between coated and uncoated implants.

Metal allergy has been proposed as a potential cause of residual pain after TKA, however, determining causality is difficult. TKA implants are typically alloys composed of several metals, and a release of ions in the joint may be responsible for the development of synovial pathologies, premature wear, or loosening, even in the absence of metal-on-metal contact in TKA. Contact dermatitis linked to Chrome and Cobalt allergies are uncommon [24, 25]. However, Nickel hypersensitivity is more common (about 25% of the population, mostly women) but may not induce cutaneous symptoms [15, 26].

TiN-coated prostheses have been developed for the theoretical mechanical advantage of ceramic coatings, allowing for superior implant hardness, high wear resistance, and lower friction [27]. Several studies have reported minimal signs of surface delamination, scratching, or coating failure within laboratory studies [28] and retrieval studies [11, 29], whereas other studies have reported wear and degradation, especially in mobile bearing tibial inserts [30]. It has also been suggested that ceramic surfaces could be more resistant to biofilm formation and reduce the risk of chronic periprosthetic joint infections. However, this theoretical advantage has not been observed in registry studies [18, 31]. The TiN surface is also thought to facilitate interdigitation between the implant and the bone cement [7].

There were no revisions and no radiological signs of wear or loosening in either group in this study, however, the follow-up period is relatively short. The estimated survival rate in the TiN group is slightly lower than other published series [8, 17, 32], however, it is likely explained by the small size of the TiN group (*n* = 14) and there was only one case of revision. Considering revisions for coating-related causes, the survival rate was 100% in the TiN group with more than 5 years of follow-up. Long-term results are required to determine the longevity of the current results.

Several published studies examined outcomes of TiN-coated mobile-bearing (MB) and fixed-bearing (FB) TKAs, reporting no differences in postoperative outcomes and survival rates [8, 33]. Postler et al. recently compared TiN-coated versus uncoated fixed-bearing TKAs and found no difference in clinical outcomes [34]. Metal ion concentrations of chromium, cobalt, and nickel at one year revealed lower levels of metal ions in the coated TKA group.

In the current study, fully cemented components were used. Thienpont similarly compared 38 patients with cemented titanium niobium nitride knee implants including 36 patients who presented with a history of nickel allergy and 2 with a chrome and cobalt allergy. Like the current study, the authors also used a 1:2 ratio to match groups of conventional chrome cobalt cemented implants with no history of metal allergy. In accordance with our results, after a mean 2-year follow-up, there was no difference in clinical outcomes and radiological measurements with a posterior-stabilized fixed bearing design [15]. Similar results have also been reported with uncemented implants: Van Hove et al. compared uncemented TiN-coated TKAs to CoCrMo TKAs with the same mobile bearing design and found no difference in postoperative pain and revision rate after 5-year follow-up [9]. Louwerens et al. found similar results after a follow-up of 10 years with uncemented mobile-bearing prostheses in a double-blinded randomized controlled trial [16]. It should be noted that the revision rate was high but comparable between the groups (6% vs. 8% for coated vs. uncoated respectively).

One of the advantages of the TiN-coated implant utilized in this study is that the design was identical in geometry to the CoCrMo prosthesis used in the control group. This eliminates the need for surgeons to undergo a learning curve when using this prosthesis for relatively rare cases of patients with suspected metal allergies.

There are several limitations to our study. Firstly, it is a retrospective study. The inherent biases to this design are however limited by using data from a prospectively collected database. Secondly, not all the patients scheduled for a TiN-coated prosthesis underwent patch metal allergy testing. The sensitivity of these skin tests is very high but the prevalence of metal allergy, especially nickel hypersensitivity, is also high. Consequently, having a positive patch test does not predict a high probability of the risk of a localized or generalized allergic reaction. Some authors now recommend lymphocyte transformation (or stimulation) tests, which measure the proliferative response of the lymphocytes after metal exposure, however, the value of such tests in the assessment of patients with suspected metal hypersensitivity remains unclear [35]. Thirdly, it would have been interesting to determine plasma metal ion concentrations of cobalt and chromium in patients of both groups. However, this was not the main objective of the study, and joint aspiration in prosthetic joints is only performed when deemed absolutely essential due to the risk of periprosthetic joint infection.

## Conclusion

TiN-coated TKA with mobile bearing resulted in satisfactory clinical outcomes, and a low revision rate, and there was no complication related to the coated implant. The use of TiN-coated prostheses in case of confirmed or suspected metal allergy provides satisfactory short-term clinic outcomes. Future long-term studies are required to confirm the longevity of these results.
